# Efficacy of Liuzijue in Improving Respiratory Muscle Dysfunction in Chronic Obstructive Pulmonary Disease: Protocol for a Randomized Controlled Trial

**DOI:** 10.2196/91283

**Published:** 2026-08-10

**Authors:** Yiting Ni, Wei Feng, Wangtao Cai, Yuxin Sun, Min Cao, Juhan Chen, Jian Li

**Affiliations:** 1 School of Rehabilitation Science, Shanghai University of Traditional Chinese Medicine, 1200 Cailun Road, Pudong New Area, Shanghai, 201203, China, 1 21-5132-2259, 1 21-5132-2257; 2 Department of Rehabilitation, Changhai Hospital, Shanghai, China

**Keywords:** Liuzijue, chronic obstructive pulmonary disease, respiratory muscle dysfunction, randomized controlled trial, clinical protocols

## Abstract

**Background:**

Chronic obstructive pulmonary disease (COPD) is a prevalent chronic lung disease, and respiratory muscle dysfunction is one of its key pathogenic mechanisms. Liuzijue, a traditional Chinese health exercise, has been widely applied in COPD rehabilitation, showing benefits in improving pulmonary function and quality of life. However, clinical evidence regarding its specific effects on respiratory muscle function remains insufficient.

**Objective:**

This study describes the protocol of a randomized controlled trial designed to evaluate the effects of Liuzijue exercise on respiratory muscle function and functional outcomes in patients with COPD.

**Methods:**

This study adopts a randomized controlled trial design, with participants randomly assigned to either a control group or a Liuzijue group. The control group receives standardized pharmacological treatment, while the Liuzijue group receives the same pharmacological treatment combined with Liuzijue intervention. The coprimary outcome measures are the 6-minute walk test and surface electromyography of the respiratory muscles. Secondary outcome measures include pulmonary function tests and respiratory questionnaires. Exploratory outcome measures include isokinetic strength testing of the core muscle groups and kinematic analysis.

**Results:**

As of manuscript submission, this trial has received ethical approval (reference number: CHEC2025-149) and secured funding in May 2024. Participant recruitment began on August 1, 2025, and has enrolled 15 participants in the control group and 8 participants in the Liuzijue group to date. Recruitment is expected to be completed by September 2026, followed by data collection until December 2026, with data analysis planned for January 2027 and the final results expected to be prepared and submitted for publication in spring 2027.

**Conclusions:**

This study is one of the first rigorously designed clinical trials to investigate the effects of Liuzijue on improving respiratory muscle function in patients with COPD, aiming to provide reliable clinical evidence for its efficacy in the rehabilitation of respiratory muscle function in this population.

## Introduction

Chronic obstructive pulmonary disease (COPD) is a common, preventable, and treatable yet noncurable heterogeneous lung disease characterized by persistent dysfunction of the airways, alveoli, and pulmonary vasculature, leading to progressively worsening symptoms such as dyspnea, cough, and sputum production [1]. The global burden of COPD continues to rise, largely attributable to heightened exposure to air pollutants and the progressive aging of the population [2]. COPD has become a major contributor to the global disease burden and is currently the third leading cause of death worldwide [3,4]. Epidemiological data indicate that between 1990 and 2019, the growth rates of COPD prevalence and incidence in China were lower than the global average, reflecting notable achievements in COPD prevention and control during this period [5,6]. However, key indicators, including the age-standardized mortality rate, age-standardized incidence rate, and disability-adjusted life years associated with COPD in China, remain at high levels, likely due to accelerated industrialization, urbanization, and population aging. Based on the research conducted by Academician Chen Wang [7], the prevalence of COPD among Chinese adults aged 40 years and older is as high as 13.7%, with an estimated patient population exceeding 100 million. Despite notable progress in prevention and control, COPD remains a major public health challenge in China.

Respiratory muscle dysfunction is a key pathological mechanism contributing to respiratory impairment in patients with COPD. Under the combined influence of multiple pathological factors, including pulmonary hyperinflation, increased ventilatory demand, inflammation, and oxidative stress, the primary respiratory muscles, such as the diaphragm and internal intercostal muscles, are subjected to both abnormal mechanical loading and metabolic imbalance. This sustained pathological burden induces microstructural alterations, including muscle fiber type transformation and mitochondrial dysfunction, ultimately leading to muscle atrophy and reductions in muscle strength and endurance [8,9]. Such alterations not only directly impair the function of the primary respiratory muscles but also indirectly exacerbate dyspnea by diminishing ventilatory reserve capacity. This, in turn, initiates a vicious cycle of worsening dyspnea, activity limitation, and progressive loss of muscle strength, thereby markedly increasing the risk of respiratory failure and systemic sarcopenia [10,11]. A previous study [8] has demonstrated a significant association between respiratory muscle strength and disease progression in COPD. Specifically, in the early stages of the disease (Global Initiative for Chronic Obstructive Lung Disease [GOLD] stages I-II), patients mainly present with respiratory muscle dysfunction, such as weakness of the internal intercostal muscles, while in the advanced stages (GOLD stages III-IV), the function of the primary inspiratory muscles, particularly the diaphragm, becomes significantly impaired [12]. Therefore, improving respiratory muscle function has become one of the key targets in the clinical rehabilitation of patients with COPD.

Clinically, common approaches to improving respiratory muscle function include pharmacological treatment, physical factor therapy, and respiratory muscle strength training. Although evidence from a study [13] demonstrating the benefits of targeted training supports the potential of these approaches to improve respiratory muscle function in COPD, it must be acknowledged that their overall impact is substantially limited by inherent methodological shortcomings. Pharmacological interventions, such as carbonic anhydrase inhibitors, while enhancing respiratory drive, may induce or exacerbate respiratory acidosis [14]. Physical modality therapy requires specialized equipment and professional operation, making clinical applications cumbersome and limiting patient adherence to long-term treatment [15]. Moreover, improper load control during respiratory muscle strength training may lead to adverse events such as headache, coughing, and muscle pain [16]. Therefore, there is a need to explore safer, more effective, and better-tolerated respiratory muscle training programs to further alleviate COPD-related symptoms and improve quality of life. In recent years, traditional Chinese health-preserving exercises have been widely used as nonpharmacological interventions in COPD rehabilitation, including Tai Chi, Baduanjin, Wuqinxi, and Yijinjing, which are notable for their simplicity, safety, and minimal risk of adverse effects [17]. Through a combination of physical movements, breathing regulation, and psychological adjustment, these exercises reflect a holistic rehabilitation concept. Existing studies [18-21] suggest that such exercises not only improve pulmonary ventilation and respiratory muscle strength in patients with stable COPD but also contribute to enhanced quality of life. Consequently, Chinese exercises have become an important adjunctive treatment for patients with stable COPD, offering broad clinical application prospects.

Liuzijue, an important component of traditional Chinese medicine for health preservation and care, is a health-promoting practice that integrates specific breathing techniques, vocalization, and physical movements, and demonstrates characteristics comparable to medical aerobic exercises commonly used in Western rehabilitation medicine, thereby being regarded as a form of aerobic gymnastics. Its technical characteristics involve controlled breathing patterns generated through specific pronunciation, supplemented by coordinated physical movements, aiming to regulate breathing, strengthen the body, harmonize the Qi and the blood, and promote mind-body balance [22,23]. This practice systematically integrates core elements of modern pulmonary rehabilitation, including respiratory muscle training, exercise training, and cardiopulmonary function enhancement [24]. The physical movements of Liuzijue mainly include flexion and extension of the upper limbs, torso rotation, chest expansion, and contraction of abdominal muscle groups. Existing clinical studies [25,26] have demonstrated that Liuzijue significantly improves respiratory function in patients with chronic respiratory diseases, particularly those with stable COPD. Liuzijue effectively alleviates dyspnea, improves lung function, enhances exercise capacity, and ultimately promotes daily functional ability and quality of life in patients through the combination of controlled vocalization and breathing with low-intensity aerobic limb movements. These improvements are typically reflected in increased expiratory volume, improved ventilation efficiency, enhanced exercise endurance, and higher health-related quality of life scores [27-29].

In addition to improving lung function, Liuzijue also exerts a positive effect on skeletal muscle, particularly the function of large muscle groups. Existing studies have shown that Liuzijue, through the pronunciation of the six characters “xu,” “he,” “hu,” “si,” “chui,” and “xi,” can simulate the physiological effects of pursed-lip breathing and diaphragmatic breathing, thereby effectively alleviating dyspnea in patients with COPD [24,30,31]. Moreover, the vocalization process during the practice prolongs exhalation time, which helps improve gas retention. Simultaneously, its coordinated limb movements constitute a low-intensity aerobic exercise model. Through the combined effects of rhythmic limb movement and large muscle group coordination training, it enhances the strength and endurance of major muscle groups such as the quadriceps and biceps [32]. However, despite the demonstrated benefits of Liuzijue in improving respiratory function and enhancing skeletal muscle strength, research on its direct effects on respiratory muscle function remains relatively limited and mostly confined to theoretical discussions. Existing studies [24,30-32] have primarily focused on improving core muscle function, with insufficient clinical evidence regarding its specific impact on respiratory muscle groups such as the diaphragm and intercostal muscles. Nevertheless, Liuzijue still holds potential for improving respiratory muscle function. Its unique combination of breathing control and limb movements may provide effective training stimuli for respiratory muscle groups. However, standardized assessment methods for respiratory muscle strength and biomechanical measurement tools are still limited in this field. In recent years, several studies [33,34] have begun to adopt novel biomechanical analysis techniques, such as isokinetic dynamometers and kinematic analysis, to more accurately quantify muscle movement patterns, providing technical possibilities for the precise evaluation of respiratory muscle function. Nevertheless, the mechanisms by which Liuzijue influences respiratory muscle function remain unclear and require further investigation to establish solid scientific evidence.

Given this background, this study was designed as a randomized controlled clinical trial to systematically evaluate the effects of the traditional Chinese health-preserving practice Liuzijue on respiratory muscle dysfunction in patients with COPD, and to further determine whether improvements in respiratory muscle performance translate into enhanced lung function, thereby fulfilling key objectives of pulmonary rehabilitation in COPD. Additionally, this study assesses the safety of Liuzijue in augmenting overall respiratory efficiency. The anticipated findings are expected to provide practical guidance for the development of clinical pulmonary rehabilitation protocols and offer a theoretical foundation for the application of Liuzijue in the management of respiratory diseases.

## Methods

### Research Design

This study is a randomized controlled clinical trial using a parallel design and evaluator blinding to investigate the effects of Liuzijue on respiratory muscle function in patients with stable COPD. Participants will be recruited from Shanghai Changhai Hospital. All patients will be in a stable phase, either receiving outpatient treatment or following hospitalization. A total of 76 clinically stable patients with COPD will be enrolled in this trial. Before the start of the intervention, respiratory physicians will perform diagnostic assessments to confirm eligibility. Eligible participants will subsequently meet with the research team to receive comprehensive information about this study’s objectives and procedures, and will be given the opportunity to ask questions. Subsequently, participants will sign the informed consent form and will then be randomly assigned to either the control group or the Liuzijue group in a 1:1 ratio.

This trial is conducted in accordance with the 2023 GOLD recommendations [35] and adheres to the diagnostic and evaluation criteria described in *Chronic Obstructive Pulmonary Disease: Diagnosis and Management* [36]. The current trial protocol has been approved (CHEC2025-149) by the Shanghai Changhai Hospital Medical Ethics Committee and has been registered with the Chinese Clinical Trial Registry (ChiCTR2500107043).

**Figure 1.**
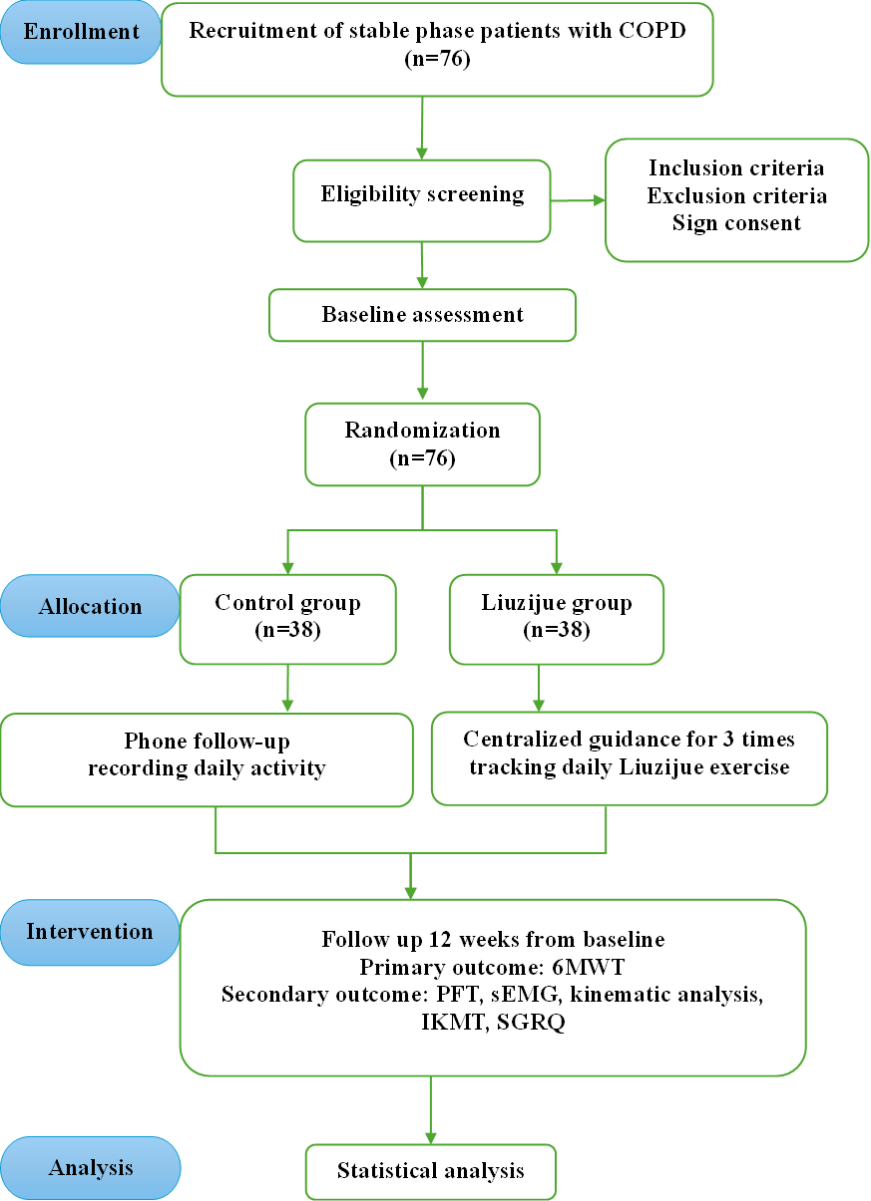
Flowchart of this trial's design and participant timeline. Eligible patients with stable COPD (GOLD stage II-III) were randomly allocated (1:1) to receive standardized pharmacological treatment alone or combined with a 12-week Liuzijue exercise intervention. Outcomes were assessed at baseline and post intervention, including 6MWT, sEMG, PFT, SGRQ, IKMT, and kinematic analysis. 6MWT: 6-minute walk test; COPD: chronic obstructive pulmonary disease; GOLD: Global Initiative for Chronic Obstructive Lung Disease; IKMT: isokinetic muscle strength test; PFT: pulmonary function test; sEMG: surface electromyography; SGRQ: St. George’s Respiratory Questionnaire.

The total duration of this study is 9 months. The flowchart of this trial’s design is shown in Figure 1, and the details of patient recruitment, group allocation, interventions, and outcome measures are summarized in Table 1.

**Table 1.** Schedule of enrollment, interventions, and assessments according to the SPIRIT^a^ guidelines.

Study period	Baseline	Allocation	Intervention	Assessment
Time point (week)	-2	0	1‐12	13
Enrollment				
Eligibility screen	✓			
Informed consent	✓			
Demographics	✓			
Disease history	✓			
Combination medication	✓			
Centralized guidance	✓			
Phone follow-up	✓	✓	✓	✓
Randomization and allocation		✓		
Interventions				
Control group			✓	
Liuzijue group			✓	
Assessments				
PFT^b^		✓		✓
6MWT^c^		✓		✓
SGRQ^d^		✓		✓
sEMG^e^		✓		✓
IKMT^f^		✓		✓
Kinematics test		✓		✓
Risk management				
Safety assessment			✓	✓
Safety monitoring			✓	✓
Risk prevention			✓	✓
Adverse event reporting			✓	✓

a SPIRIT: Standard Protocol Items: Recommendations for Interventional Trials.

b PFT: pulmonary function test.

c 6MWT: 6-minute walk test.

d SGRQ: St. George’s Respiratory Questionnaire.

e sEMG: surface electromyography.

f IKMT: isokinetic muscle strength test.

### Participants

#### Patient Recruitment

The recruitment strategy primarily includes the use of posters, community sensitization, online promotion, and screening of patients from the respiratory outpatient department and those previously hospitalized.

We specifically targeted GOLD stage II-III patients for this study. This group gives us the best of both worlds: they have clear respiratory muscle dysfunction to detect mechanistic changes, yet are still able to safely perform the full Liuzijue protocol. We excluded stage I patients because they often lack measurable weakness, making improvement hard to detect—essentially a floor effect. Stage IV patients were excluded due to higher exacerbation risk and poor tolerance to exercise. We recognize this obviously limits generalizability to the broader COPD population. However, stage II-III is the largest and most clinically relevant subgroup in need of rehabilitation, and showing efficacy here is a logical first step before moving to more severe stages.

Before enrollment, participants will be provided with detailed information about this trial, including the research objectives, group allocation methods, intervention period, potential benefits, and possible risks. All participants must sign an informed consent form before the start of this trial to confirm their voluntary participation. Baseline assessments will then be conducted by the research team. All personal information will be anonymized and treated with strict confidentiality. It will be stored at the hospital and used exclusively for research purposes. Participants have the right to withdraw from this study at any time, and all cases of withdrawal and the corresponding reasons will be accurately documented by the research team.

#### Diagnostic Criteria

Based on the diagnostic criteria for COPD outlined in the 2023 GOLD guidelines [35] and *Chronic Obstructive Pulmonary Disease: Diagnosis and Management* [36], patients presenting with the following clinical manifestations can be diagnosed with COPD: (1) patients presenting with chronic cough, sputum production, dyspnea, wheezing, or chest tightness; (2) pulmonary function tests (PFTs) performed after inhalation of a bronchodilator showed a forced expiratory volume in one second (FEV₁) <80% of the predicted value and an FEV₁/forced vital capacity (FVC) <70%, indicating persistent airflow limitation despite medication; and (3) according to the GOLD guidelines, COPD being classified into four severity stages: I, II, III, and IV, based on the degree of airflow limitation and symptom severity.

#### Inclusion and Exclusion Criteria

Participants will be screened according to the predefined eligibility criteria before enrollment. Detailed inclusion and exclusion criteria are presented in Textbox 1.

Textbox 1. **Inclusion and exclusion criteria for participant eligibility.**

Inclusion criteria

- Diagnosed with stage II moderate chronic obstructive pulmonary disease (COPD) and stage III severe COPD.
- Maintained a stable condition for at least 12 weeks before randomization, with symptoms of cough, sputum production, and dyspnea remaining stable or mild, and no acute exacerbations occurring during this period.
- No gender restrictions; aged between 40 and 80 years.
- Participants who are willing to participate in this trial, can cooperate actively, and demonstrate good compliance.
- Patients who have not engaged in any form of regular traditional Chinese health-preserving exercises—defined as at least 30 minutes per session, twice daily, five times per week—during the six months before this trial.

Exclusion criteria

- Patients younger than 40 years or older than 80 years.
- Patients diagnosed with COPD who are experiencing an acute exacerbation requiring hospitalization or targeted pharmacological treatment.
- Patients who have participated in any structured pulmonary rehabilitation program, regular exercise training, or traditional Chinese health exercise programs within the 12 months before screening.
- Patients with COPD complicated by other respiratory diseases, such as asthma, pulmonary infections, tuberculosis, bronchiectasis, or lung cancer, who are deemed unsuitable for rehabilitation exercises.
- Patients with COPD complicated by severe diseases in other major systems, including cardiovascular, cerebrovascular, hematologic, hepatic, renal, digestive, endocrine, or nervous systems.
- Patients unable to cooperate with treatment and assessments due to conditions such as mental disorders or impaired consciousness.
- Participants concurrently enrolled in other clinical intervention or drug trials.
- Patients with musculoskeletal disorders or open injuries that preclude limb muscle strength assessment.

### Randomization and Blinding

SPSS (version 27.0; IBM) will be used to generate a random number list. A block randomization algorithm will assign a unique random number to each participant, and this information will be kept strictly confidential. All random numbers will be sealed in opaque envelopes, and group allocation will be performed using the envelope method. After completing baseline assessments, trained researchers will randomly select envelopes to assign participants to either the control group or the Liuzijue group according to the numbers contained therein.

Given that this trial involves physical intervention, it is difficult to blind the participants. Therefore, independent outcome assessors who are not involved in participant recruitment, intervention delivery, or group allocation will be used. All outcome assessments will be conducted by these blinded assessors according to standardized procedures. In addition, data analysts will also be blinded to group allocation in order to minimize bias and enhance the reliability of the results.

If it becomes necessary to unblind the outcome assessors or data analysts, the reasons must be documented and reported in detail. During data analysis, appropriate statistical methods will be applied to control potential bias.

### Sample Size Calculation

The sample size calculation for this study is performed using SPSS (version 27.0) and was based on the primary outcome of the 6-minute walk test (6MWT). According to evidence from previously published randomized controlled trials and systematic reviews in patients with COPD, the minimal clinically important difference (MCID) for 6MWT is approximately 54 m, and the baseline SD is generally reported to range between 70 and 90 m. To ensure a conservative and methodologically robust estimation, an SD of 80 m will be adopted for the present study [37,38].

A two-sided significance level of *α*=.05 and a type II error rate of *β*=.20 (80% power) were specified. Based on an MCID of 54 m, the required sample size is calculated using the following formula: $n=\frac{{\left(Z_{\frac{\alpha }{2}}+Z_{\beta }\right)}^{2}\times 2\times {SD}^{2}}{{MCID}^{2}}$. This yields a minimum sample size of 32 participants per group, for a total of 64 participants across both groups. Accounting for an anticipated dropout rate of 15%, the final required sample size is adjusted to 76 participants, with 38 participants allocated to each group.

### Grouping and Intervention

#### Control Group

Clinical respiratory physicians will formulate standardized medication regimens for participants based on the 2023 GOLD guidelines [35] and *Chronic Obstructive Pulmonary Disease: Diagnosis and Management* by American Family Physician [36]. Participants will receive conventional pharmacological treatment throughout this trial. During this study, participants will be instructed to abstain from any form of regular traditional Chinese health-preserving exercises, defined as twice daily sessions of 30 minutes each, five times per week, according to a previous study [39]. To minimize potential attention and contact bias arising from differences in interaction frequency between groups, participants in the control group will attend the hospital once weekly, where this study’s team will manage musculoskeletal conditions unrelated to respiratory function (eg, neck, shoulder, low back, and lower limb pain). Meanwhile, we will restrict our interventions as follows: pain treatment will be limited to symptomatic management of common musculoskeletal issues, without any intervention aimed at improving exercise capacity, and analgesics will not be used. This arrangement is intended to ensure comparable clinical contact time and follow-up support between groups. In addition, the control group will receive at least five telephone follow-ups per week to record medication adherence, daily activity levels, and general health status. No respiratory exercise instruction or respiratory rehabilitation-related intervention will be provided during these contacts. In accordance with ethical considerations, participants in the control group will be offered free Liuzijue exercise guidance after completion of this trial.

#### Liuzijue Group

Based on the control group, the same standardized medication regimens, as prescribed by clinical respiratory physicians, will be implemented. During this trial, participants will be required to perform regular Liuzijue exercise sessions according to a prescribed regimen. In the two weeks preceding the formal intervention, participants in the Liuzijue group will receive three intensive training sessions conducted by the research team to ensure mastery of the complete movements and key technical elements of the Liuzijue method through systematic instruction. High-definition demonstration videos will be distributed via mobile devices to facilitate correct performance of the prescribed exercises at home, with daily check-ins required. Each participant in the Liuzijue group will be provided with an exercise log card, on which they will record detailed information after each session, including exercise date, time, frequency, duration, location, and perceived exertion (assessed using the Borg CR-10 [Category-Ratio 10] scale, see Table 2). Weekly exercise logs will be submitted at each intensive training session. Simultaneously, researchers will maintain a logbook documenting attendance during the concentrated interventions, collect the exercise log cards, and participants will be engaged in discussions regarding their exercise performance to ensure adherence to this trial’s protocol. During supervised group exercise sessions, trial personnel who have received standardized training will provide on-site correction of participants’ movements. The degree of movement standardization will be systematically documented in the case report forms to ensure consistency of the intervention and to minimize potential impacts on this trial’s outcomes.

**Table 2.** Borg CR-10^a^ rating of perceived exertion scale used to monitor exercise intensity during Liuzijue training sessions. Participants rated their perceived exertion on a scale from 0 ("nothing at all") to 10 ("maximal"), with the target intensity maintained between 4 and 6.

Rating	Description
0	Nothing at all
0.5	Very, very light
1	Very light
2	Light
3	Moderate
4	Somewhat hard
5	Hard
6	Hard
7	Very hard
8	Very hard
9	Very, very hard
10	Maximal

a CR-10: Category-Ratio 10.

**Figure 2.**
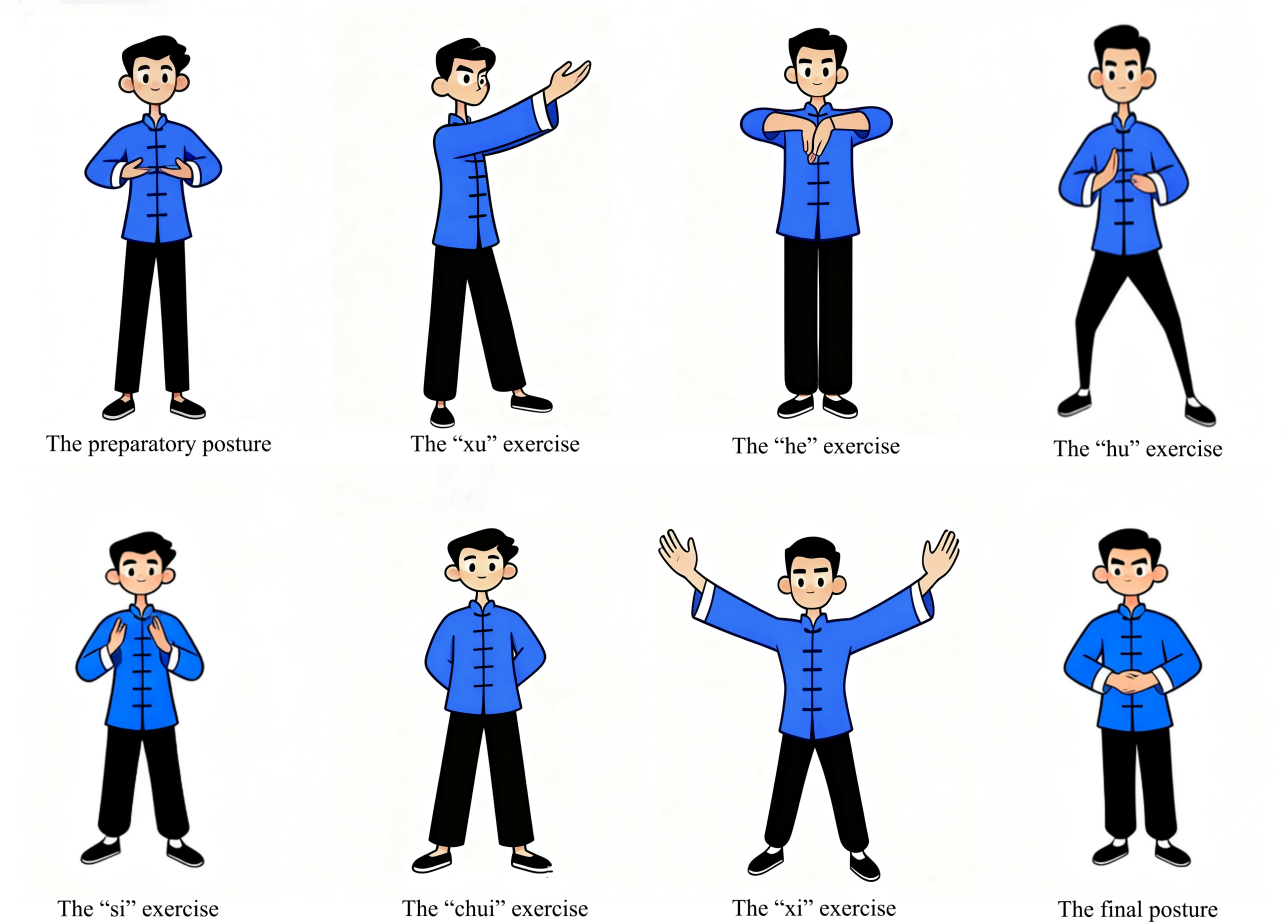
Illustration of the Liuzijue exercise routine. The routine consists of eight components: a preparatory posture, six-character vocalizations (“xu,” “he,” “hu,” “si,” “chui,” and “xi”), and a closing posture. Each session lasts approximately 30 minutes and is performed twice daily, 5 days per week, for 12 weeks.

The Liuzijue exercise prescription primarily comprises eight components: the preparatory posture, the “xu” exercise to calm the liver Qi, the “he” exercise to replenish the heart Qi, the “hu” exercise to cultivate the spleen Qi, the “si” exercise to replenish the lung Qi, the “chui” exercise to replenish the kidney Qi, the “xi” exercise to regulate the Sanjiao, and the final position [40]. The Liuzijue exercise is illustrated in Figure 2. Each session lasts approximately 30 minutes and is performed twice daily, five days per week. One day per week is dedicated to supervised, intensive practice at the hospital under the guidance of a rehabilitation therapist, while the remaining six days involve home-based practice using Liuzijue instructional videos. The intervention period lasts 12 weeks.

The intervention will consist of performing two consecutive rounds of the command-version Liuzijue routine issued by the General Administration of Sport of China. All movements will be required to be executed slowly, continuously, smoothly, and with lightness, in precise coordination with the breathing rhythm—inhaling during the upward movement of the upper limbs and exhaling during their descent. The entire practice emphasizes the integration of breath regulation, body posture, and mindful concentration, with participants instructed to maintain relaxed shoulders, a stable core, and natural joint mobility. A single round of Liuzijue requires approximately 15 minutes, resulting in a total intervention duration of about 30 minutes per session. Throughout the practice, attention will be given not only to the accuracy of the movements but also to the synchronization of breathing patterns, thereby maximizing its rehabilitative benefits for the respiratory system.

Participants will be instructed by the research team to take short breaks if they experience fatigue, exhaustion, or dyspnea during exercise, and to resume once symptoms subside. Exercise intensity will be maintained between 4 and 6 on the Borg CR-10 Dyspnea Rating Scale (detailed scoring in Table 2). During exercise sessions, participants’ heart rates will be monitored with the target exercise intensity set at 60%‐80% of each participant’s age-predicted maximum heart rate.

### Outcome Measurement

The outcome measurements for both groups and the specific assessment time points are detailed in Table 1.

### Primary Outcome Measures

#### Overview

The primary outcomes of this trial are surface electromyography (sEMG) and 6MWT.

#### sEMG

sEMG is a noninvasive technique that allows real-time and precise assessment of the electrical activity of superficial muscles, thereby providing an objective reflection of muscle recruitment and functional status. Previous studies [41,42] have demonstrated a high correlation between sEMG and invasive electromyography (EMG) signals, while effectively avoiding the stress responses induced by traditional invasive methods, which facilitates the acquisition of more reliable experimental data.

In this study, a 16-channel NORAXON Wireless EMG system (NORAXON Wireless EMG) combined with EMGworks Acquisition 4.5 (Delsys) software will be used to noninvasively evaluate changes in respiratory muscle strength in patients with COPD before and after the intervention. The primary parameters measured will include the root mean square of the EMG signal, averaged EMG, and mean power frequency. Root mean square and averaged EMG are used to quantify the intensity of muscle activation, while mean power frequency is used to assess muscle fatigue [43]. The muscles monitored include the sternocleidomastoid, trapezius, intercostal muscles, diaphragm, rectus abdominis, external oblique, internal oblique, transversus abdominis, and multifidus. Electrode placement strictly follows the SENIAM (Surface Electromyography for the Non-Invasive Assessment of Muscles) protocol, with recommended locations for respiratory muscles summarized in Table 3 [43-47]. Before signal acquisition, the skin will be thoroughly cleansed with alcohol to reduce impedance and ensure high-quality data.

**Table 3.** Electrode placement locations for respiratory muscles based on the SENIAM^a^ protocol.

No.	Muscle	Electrode placement
1	Sternocleidomastoid	The midpoint of the prominently bulging muscle on the anterolateral neck when the head is turned to the opposite side
2	Trapezius	The midpoint between C7^b^ and the acromion
3	Intercostal muscles	Two finger-breadths lateral to the sternum, at the midpoint between the second and third ribs
4	Diaphragm	The intersection of the midclavicular line and the 8th rib
5	Rectus abdominis	Two finger-breadths lateral to the umbilicus
6	External oblique	The intersection of the anterior axillary line and the 10th rib
7	Internal oblique	Located 2 cm superior to the ASIS^c^
8	Transversus abdominis	2 cm medial to the ASIS, slightly below the horizontal line of the umbilicus
9	Multifidus	The junction between the middle $\frac{1}{3}$ and medial $\frac{1}{3}$ of the line from PSIS^d^ to L5^e^

a SENIAM: Surface Electromyography for the Non-Invasive Assessment of Muscles.

b C7: the seventh cervical vertebra.

c ASIS: anterior superior iliac spine.

d PSIS: posterior superior iliac spine.

e L5: the fifth lumbar vertebra.

**Figure 3.**
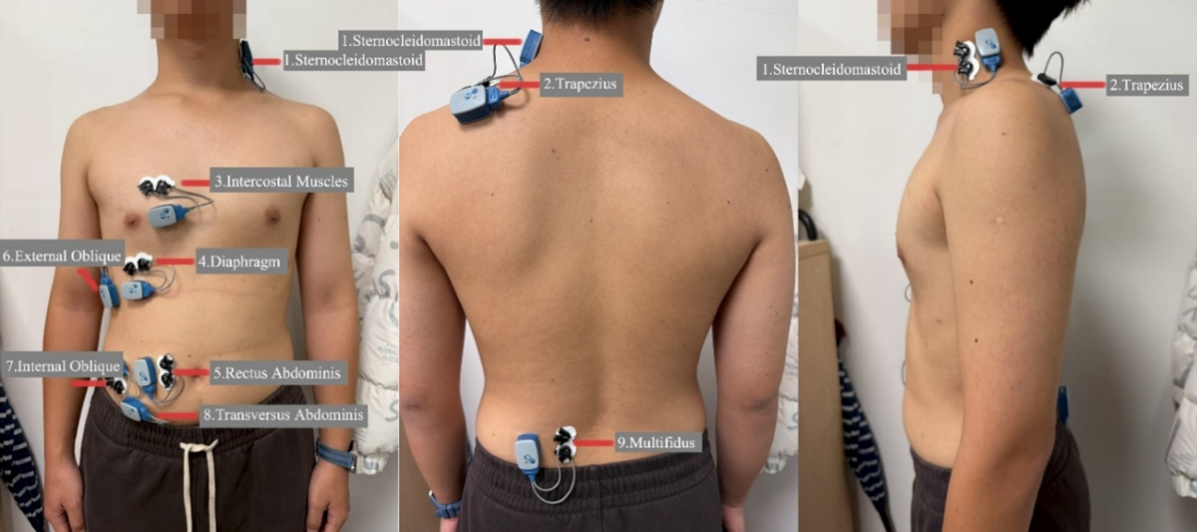
Electrode placement for surface electromyography assessment of respiratory muscles. Electrodes were placed on the sternocleidomastoid, trapezius, intercostal muscles, diaphragm, rectus abdominis, external oblique, internal oblique, transversus abdominis, and multifidus muscles according to the SENIAM (Surface Electromyography for the Non-Invasive Assessment of Muscles) recommendations.

The sEMG assessment will consist of static and dynamic tests. In the static test, participants will stand quietly while EMG signals are recorded for 5 seconds at rest, at the end of maximal inspiration, and at the end of maximal expiration, with each condition repeated three times at two-minute intervals. The dynamic test will involve a 10-meter walking trial, during which participants are instructed to walk as fast as possible along a corridor while both walking time and EMG signals are recorded simultaneously. The electrode placement for sEMG is illustrated in Figure 3. The sEMG data will be collected in week 0 and week 13.

#### 6MWT

6MWT is a widely used method for evaluating exercise capacity in patients with respiratory disorders and for assessing the efficacy of clinical interventions. Previous studies [48,49] have demonstrated that the 6MWT serves as a valid and reliable measure of functional capacity and health-related quality of life in patients with COPD, while also providing an indicator of disease severity. Based on previous findings [37,38], the MCID for 6MWT ranges from 30 to 54 m, and the baseline SD is between 70 and 90 m. This study will use a 6MWT data acquisition system in combination with a dynamic vital signs monitor, CB200 (Contec), for testing. Participants will be instructed to walk back and forth along a 30-meter flat corridor at their maximum tolerable speed for six minutes. Distance markers will be placed every 5 meters to aid measurement. The recording device will capture the total walking distance as well as heart rate and oxygen saturation before and after the test. The 6MWT outcome will serve as a primary indicator of intervention efficacy, assessed in week 0 and week 13.

### Secondary Outcome Measures

#### Overview

The secondary outcomes of this trial include PFT, St. George’s Respiratory Questionnaire (SGRQ) and isokinetic muscle strength test (IKMT) of the core muscle group.

#### PFT

PFT is widely recognized as the gold standard for the diagnosis and clinical efficacy assessment of COPD. A previous study [50] has demonstrated that PFT not only provides precise measurements of multiple pulmonary function parameters at rest but also serves as a reliable predictor of long-term prognosis. In this study, PFT will be performed by experienced respiratory technicians in the Pulmonary Function Laboratory of Shanghai Changhai Hospital using a Masterscreen-PFT system (Jaeger), strictly following the standardized procedures recommended by the 2021 ATS/ERS guidelines [51]. Before testing, participants will inhale salbutamol, and measurements will be conducted 25 minutes postinhalation [52]. During testing, participants will be instructed to perform forced exhalations lasting at least six seconds per maneuver, followed by brief rest intervals, and to repeat the maneuver until at least three acceptable measurements are obtained. Recorded parameters include FVC, FEV1, the FEV1-to-FVC ratio (FEV1/FVC), percentage of predicted FEV1 (forced expiratory volume in one second percentage predicted), percentage of predicted FVC (forced vital capacity percentage predicted), and the percentage value of FEV1/FVC (FEV1/forced vital capacity percentage). PFT assessments will be performed in week 1 and week 13.

#### SGRQ

SGRQ is a simple, effective self-administered instrument widely used for the assessment of respiratory diseases, including chronic bronchitis and COPD [53]. A previous study [54] has demonstrated a strong correlation between SGRQ scores and changes in COPD severity, making it a valuable tool for evaluating disease status and predicting prognosis. The total score ranges from 0 to 100, with higher scores indicating poorer health-related quality of life. Based on prior evidence [55,56], a reduction of at least four points in the total score was considered the MCID. In this trial, the SGRQ will be administered at baseline and post intervention. Paper-based questionnaires will be collected and analyzed in a standardized manner to generate total scores and three component scores—symptoms, activity, and impacts. Assessments will be conducted by trained research team members in week 0 and week 13.

#### IKMT of the Core Muscle Group

**Figure 4.**
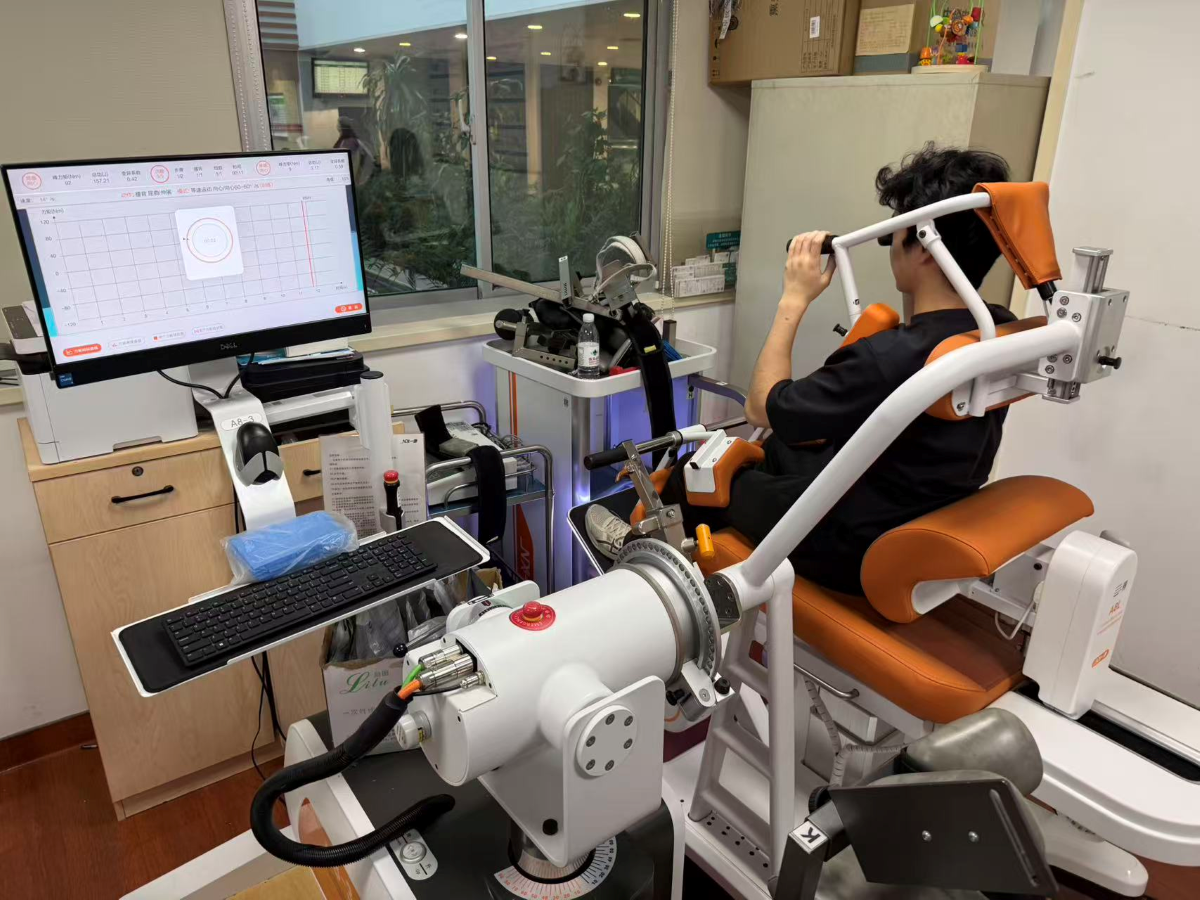
Isokinetic muscle strength test of the core muscle group. Participants were seated with the trunk flexion-extension axis aligned with the level of the greater trochanter. The pelvis, lower extremities, and shoulders were stabilized during testing. Isometric trunk flexion and extension strength were assessed using the IKANG multijoint muscle training and testing system.

IKMT combines the methodological strengths of isometric and isotonic assessments, allowing for precise quantification of muscle strength with excellent reproducibility and reliability [57]. Considering the critical role of core musculature in respiratory assistance, IKMT-based evaluation of core muscle function offers valuable insights into respiratory muscle status. In this trial, an IKANG multijoint muscle training and testing system (NX, A8-3, CHN) will be used to perform isometric assessments of trunk core muscles to examine functional changes induced by intervention. In accordance with established studies [34,58-61], the isometric contraction mode was applied. During testing, participants will be seated in the standardized position recommended by the manufacturer, with the trunk flexion–extension axis aligned at the level of the greater trochanter. The pelvis, lower extremities, and shoulders will be firmly stabilized with fixation devices. Standardized calibration will be performed before testing, and all assessments will be conducted by the same evaluator within a consistent time frame to reduce variability. Each participant will complete three warm-up repetitions followed by five maximal-effort isometric contractions for both trunk flexion and extension. The primary outcome variables include peak torque, peak torque to body weight ratio, time to peak torque, and average power. The IKMT of the core muscle group is illustrated in Figure 4. All measurements will be obtained in week 0 and week 13.

### Exploratory Outcome Measures

#### Overview

We consider gait and plantar pressure parameters as exploratory outcomes. These measures do more than just provide descriptive biomechanical data—they also help us rule out a plausible alternative: that any improvement in 6MWT could be driven primarily by enhanced lower limb function rather than by respiratory muscle gains. Should these parameters show meaningful changes, we will include them as covariates in the 6MWT analysis, which allows us to tease out the independent contribution of respiratory muscle function.

#### Kinematics Test

The kinematic test uses optical, pressure, and other sensor technologies to quantitatively evaluate the activity of the human musculoskeletal system, thereby elucidating the biomechanical patterns of human movement. In this study, gait analysis and plantar pressure assessment will be conducted in patients with stable COPD to investigate lower limb functional improvements following Liuzijue exercise intervention.

**Figure 5.**
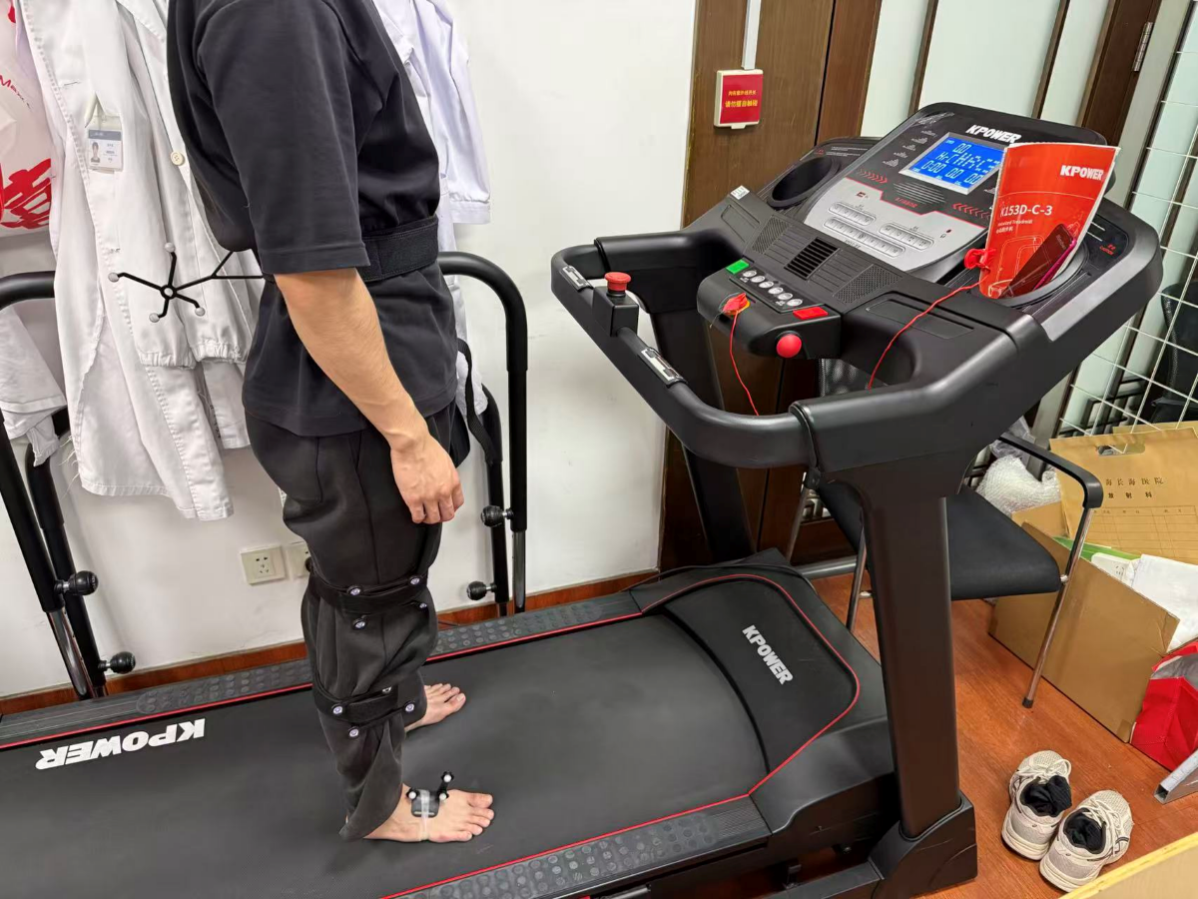
Gait analysis using a joint motion function testing system. Reflective markers were attached to the participants’ waist, femur, tibia, and foot. Participants walked forward and backward on a treadmill at a speed of 3 km/h for 15 seconds in each direction. Hip, knee, and ankle kinematics, as well as overall gait stability, were evaluated.

A previous [62] study had demonstrated that gait analysis can sensitively reflect the disease status of patients with COPD and predict their functional prognosis. In this study, a joint motion function testing system (innoMotion, CHN) will be used in combination with a treadmill system (KPOWER, K153D-C-3, CHN) and dedicated gait analysis software (Xmotion, CHN) to evaluate gait characteristics before and after the intervention. The primary outcome measures include joint kinematics of the hip, knee, and ankle, as well as overall gait stability. Before testing, reflective markers will be attached to the participants’ waist, femur, tibia, and foot, after which they will stand on the treadmill. During the test, participants first walk forward at a speed of 3 km/h, and 15 seconds of gait data will be collected; they then walk backward at the same speed, and another 15 seconds of data will be recorded. The gait analysis is illustrated in Figure 5.

**Figure 6.**
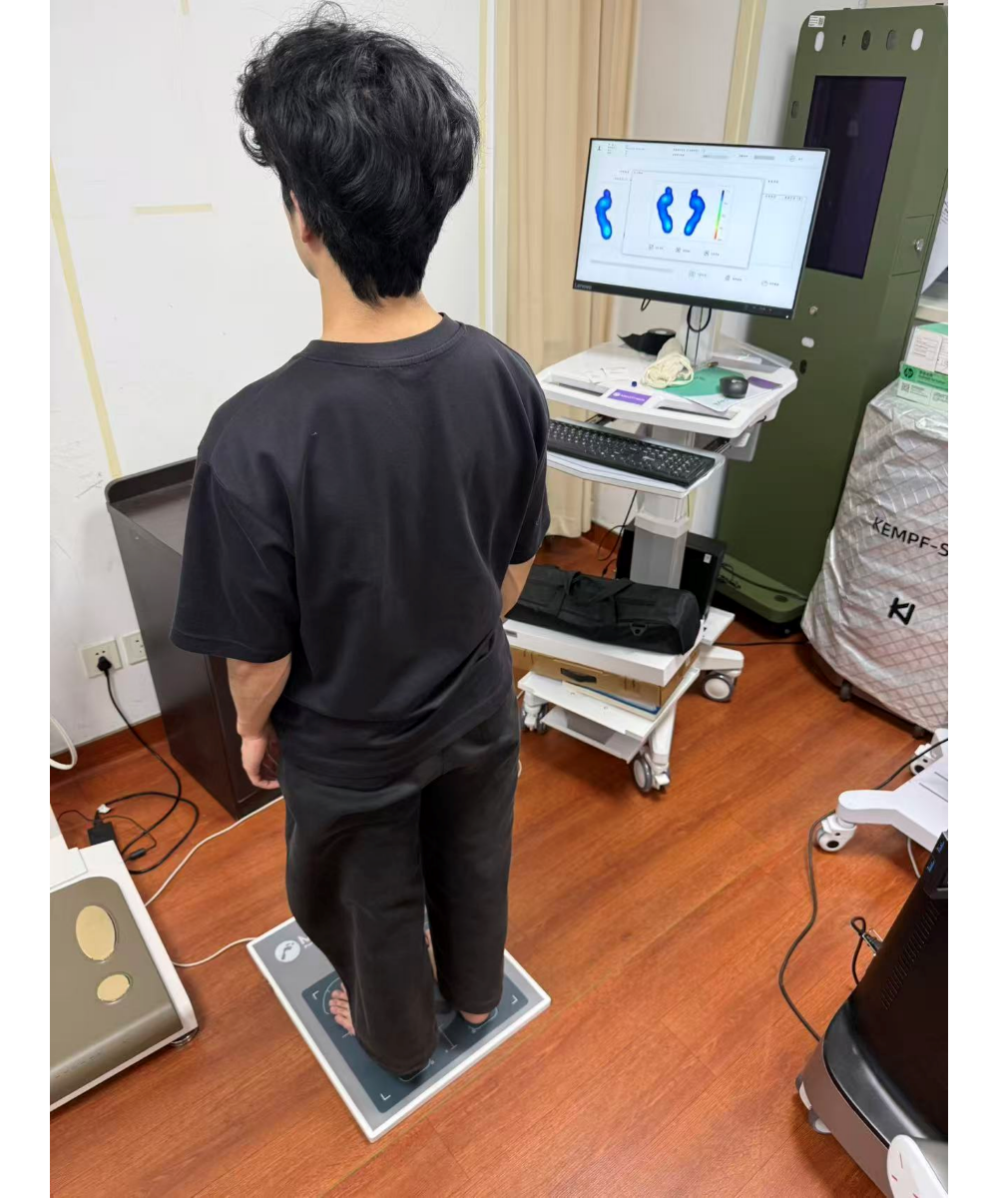
Plantar pressure assessment using a pressure testing platform. Participants stood and walked on the pressure testing platform to assess foot arch characteristics, inversion/eversion patterns, and plantar force distribution using the gait training and testing software.

Plantar pressure assessment enables accurate evaluation of the medial longitudinal arch and the distribution of plantar forces during walking. Evidence suggests that patients with COPD may present with abnormal plantar pressure patterns, potentially contributing to a decline in quality of life [63]. In this study, a plantar pressure testing platform (Medtrack, CHN) in conjunction with the gait training and testing software (version 5.1.0.8) will be used for analysis. The system is used to record and evaluate the condition of the foot arch and the inversion/eversion curves, thereby assessing changes in plantar pressure before and after the Liuzijue exercise intervention. The plantar pressure testing is illustrated in Figure 6.

Both gait analysis and plantar pressure assessment will be performed in week 0 and week 13.

### Safety Assessment

A rigorous adverse event monitoring system will be implemented throughout this trial. All adverse events occurring during this study will be recorded in detail, including the time of onset, clinical manifestations, severity, potential causes, and management measures. In the event of an adverse reaction, the clinical investigators will assess the participant’s condition and determine whether continuation in this trial is appropriate. Necessary medical care will be provided, and all serious adverse events will be reported promptly to the Medical Research Ethics Committee of Shanghai Changhai Hospital.

### Statistical Analysis

Statistical analyses will be executed using SPSS (version 27.0), adhering to a two-sided significance threshold of *P*<.05 while reporting effect sizes and 95% CIs where appropriate. Baseline characteristics will be summarized solely through descriptive statistics without formal hypothesis testing, with continuous variables first evaluated for normality and homogeneity of variance to determine their presentation as either mean with SD or median with IQR.

Under an intention-to-treat framework encompassing all randomized participants, missing data patterns will be evaluated and addressed via multiple imputations, the robustness of which will be subsequently verified through sensitivity analyses including per-protocol and complete-case approaches. Primary outcomes will be modeled using linear mixed-effects specifications that will incorporate group, time, and their interaction as fixed effects alongside participant-level random intercepts to account for the repeated-measures design.

For continuous secondary outcomes, analysis of covariance adjusted for baseline values will be used where appropriate, whereas other continuous and categorical variables—the latter summarized as frequencies and percentages—will be scrutinized using parametric, nonparametric, chi-square, or Fisher exact tests depending on data distribution, with false discovery rate adjustments applied throughout to mitigate type I error inflation from multiple comparisons.

### Compliance Assessment

Adherence in the Liuzijue group will be assessed based on attendance at the weekly hospital-based supervised training sessions and completion of exercise log records. Participants who complete at least 85% of the prescribed exercise sessions will be classified as having good adherence.

In the control group, adherence will be evaluated through participation in regular telephone follow-ups, which will include confirmation of medication compliance, verification of no disease-related medication adjustments, and confirmation of abstention from traditional Chinese health exercises. Participants who complete at least 85% of the scheduled follow-up calls and continue to meet the inclusion and exclusion criteria will be classified as having good adherence.

### Quality Control

This research protocol has been revised and reviewed by multidisciplinary experts in respiratory medicine and methodology, with standard operating procedures established before trial initiation. Throughout this study, all research team members will undergo standardized training to ensure thorough familiarity with the protocol and specific operational procedures. Participant enrollment will remain consistent throughout this trial, and uniform standards will be applied to all outcome assessments to ensure data accuracy and reliability.

### Data Collection and Management

Standardized data collection forms will be developed before this study, incorporating all required variables and outcome measures. Research team members will record and collect data following consistent protocols. All study data will be securely stored in a dedicated database for long-term preservation. Upon study completion, all data and associated documentation will be archived for potential future review.

In the event of missing data, efforts will be made to identify the reasons for data loss and to retrieve missing information whenever possible. The extent and patterns of missing data will be documented and reported. Appropriate statistical methods will be applied to handle missing data during analysis, based on the nature and proportion of missing values, to minimize potential bias and ensure the robustness of this study’s results.

### Data Monitoring

#### Composition of Data Monitoring Committee

As the intervention in this trial is noninvasive and carries minimal risk of physical harm, an independent Data Monitoring Committee will not be established. The safety, compliance, and data integrity will be jointly monitored by the research team and the Medical Ethics Committee of Shanghai Changhai Hospital.

If this trial cannot be continued for any reason, the principal investigator reserves the right to terminate this study. All data collected up to that point will be securely retained by the principal investigator until this trial is resumed.

Given the low-risk nature of the intervention, no interim analysis is planned.

#### Auditing Trial Conduct

This trial will be audited monthly by rotating investigators, with verification conducted by the principal investigator. The audit will cover data integrity, patient adherence, and the reporting of adverse events.

### Patient and Public Involvement

Patients and members of the public were not directly involved in the design, implementation, data analysis, or manuscript preparation of this study. We understand and value the role of patient and public involvement in clinical research; however, due to the nature and feasibility of this study, their participation was not included in this research process.

### Ethical Considerations

This study has been approved (CHEC2025-149) by the Shanghai Changhai Hospital Medical Ethics Committee. Any unexpected adverse events will be recorded and reported to the ethics committee according to institutional guidelines.

## Results

This trial is supported by the Traditional Chinese Medicine Research Project of the Shanghai Municipal Commission of Health (No. 2024QN094), which was awarded in May 2024. Participant recruitment commenced in August 2025. As of submission of this manuscript, 23 participants had been enrolled, including 15 participants in the control group and 8 participants in the Liuzijue group. Data collection is expected to be completed by late 2026, followed by statistical analysis in early 2027. This study’s protocol (version 1.1) commenced participant recruitment on August 1, 2025. Recruitment is expected to be completed by September 2026, followed by data collection through December 2026. Data analysis is planned for January 2027. The final trial results are anticipated to be published in spring 2027.

## Discussion

### Principal Findings

This study is expected to demonstrate that Liuzijue can improve respiratory muscle function, leading to enhanced exercise capacity and health-related quality of life in patients with stable COPD.

As the third leading cause of death worldwide, COPD profoundly impairs patients’ exercise capacity and quality of life, while imposing a substantial burden on health care systems and socioeconomic resources [3,4]. Despite the availability of various pharmacological and nonpharmacological interventions, the overall control rate of COPD remains unsatisfactory, particularly in the domain of rehabilitation during the stable phase. This highlights the need for effective rehabilitation strategies targeting both functional capacity and respiratory muscle performance.

A growing body of evidence indicates that traditional Chinese exercise therapies can significantly improve the exercise capacity and quality of life in patients with stable COPD and have been incorporated into pulmonary rehabilitation programs [64]. However, current research on Liuzijue remains limited, with a lack of mechanistic studies from the perspective of respiratory muscle function changes.

In line with this aim, the discussion is organized around functional outcomes, mechanistic evidence, and clinical implications. To systematically evaluate changes in symptoms and functional outcomes in patients with COPD before and after the intervention, this study will use three well-established and widely used outcome measures, including PFT, the 6MWT, and the SGRQ, with the 6MWT designated as the primary outcome. As a core indicator reflecting overall exercise capacity and functional performance, the 6MWT is particularly sensitive in capturing changes in daily physical activity levels among patients with COPD and is therefore selected as the primary endpoint of this study [48,49]. PFTs will be used to objectively assess the physiological status of respiratory function [50], while the SGRQ will comprehensively reflect symptom burden and health-related quality of life from the patient’s subjective perspective [53,54]. Collectively, these measures establish a comprehensive outcome evaluation framework encompassing physiological, functional, and patient-reported dimensions, in accordance with the requirements of high-quality clinical research for scientific rigor, comparability, and clinical relevance of primary and secondary outcomes.

Building upon this framework, this study further integrates static and dynamic sEMG together with IKMT to objectively characterize the functional properties of respiratory and core muscle groups. This approach aims to address a key limitation of previous COPD rehabilitation studies, in which the mechanisms underlying intervention effects—particularly those related to respiratory muscle function—have received relatively limited attention. Specifically, sEMG, as another primary outcome measure, can objectively and clearly reflect changes in respiratory muscle function in participants. In this study, both static and dynamic sEMG assessments will be performed to comprehensively evaluate respiratory muscle activation patterns under different conditions. Static sEMG will be applied to analyze muscle excitability and recruitment patterns of respiratory muscles at rest, whereas dynamic sEMG will be used to record muscle activation during movement [41-43]. Combined with IKMT of the core musculature, these assessments will enable quantitative evaluation of the strength and endurance of accessory respiratory muscles [57]. Through comprehensive multimodal analysis, the functional status and adaptive changes of respiration-related muscle groups can be more thoroughly elucidated, providing objective and reliable evidence for evaluating the mechanistic effects and long-term value of Liuzijue interventions.

In addition, a novel kinematic perspective will be introduced by incorporating gait analysis and plantar pressure assessment to systematically evaluate changes in lower-limb function. This strategy will help exclude potential confounding effects arising from muscular adaptations outside the respiratory system, thereby further strengthening the accuracy and internal validity of intervention effect determination [62,63].

The two coprimary outcomes in this study will serve distinct yet complementary purposes. The 6MWT is selected to capture clinically meaningful changes in functional exercise capacity, whereas sEMG provides a more direct assessment of neuromuscular activity in the respiratory muscles, thereby offering mechanistic insight into the intervention’s effects. Regarding sample size estimation, we based our calculations on the 6MWT, primarily because published data on sEMG responses to Liuzijue training in patients with COPD remain limited, precluding a reliable a priori estimate.

Overall, the anticipated findings are expected not only to provide high-quality evidence to support the optimization and individualization of clinical pulmonary rehabilitation programs, but also to offer a more in-depth and systematic theoretical interpretation of the mechanisms through which traditional Chinese health-preserving exercises and rehabilitation techniques exert their effects in COPD rehabilitation.

This study describes the protocol of a randomized controlled trial designed to evaluate the effects of Liuzijue exercise on respiratory muscle function and functional outcomes in patients with COPD, while further elucidating its role in improving the status of specific respiratory muscles and characterizing the recruitment patterns of major respiratory muscle groups. These findings will provide an important theoretical basis for the application of traditional Chinese health-preserving exercises in respiratory function enhancement. Building on this, this study will also focus on evaluating the therapeutic effects and safety of Liuzijue in patients with COPD-related respiratory muscle dysfunction.

Several limitations should be noted. First, this study population is limited to patients with stable COPD, excluding those with varying degrees of disease severity, which may restrict the generalizability of the findings. Second, this study has certain limitations in the assessment of upper limb muscle parameters, which may not fully capture the effects of Liuzijue exercise on the accessory respiratory muscles of the upper limbs. Third, there is currently no highly precise kinematic tool capable of directly measuring respiratory muscle changes; therefore, the kinematic parameters in this study remain limited. Finally, due to the inherent characteristics of the intervention, a double-blind trial is not feasible, which may introduce a potential risk of bias. Despite these limitations, the rigorous design and comprehensive outcome measures are expected to yield clinically meaningful evidence.

The findings of this study will be disseminated through peer-reviewed journal publications, and standardized instructional videos will be developed to facilitate learning and application among community-based patients.

### Conclusions

In summary, this randomized controlled trial aims to evaluate the effects of the Liuzijue on respiratory muscle function, with a primary focus on its efficacy and safety in patients with stable COPD. As the first clinical trial specifically investigating the impact of the Liuzijue on respiratory muscle function within this population, it addresses a significant gap in the literature. The findings are expected to deepen the understanding of the mechanisms underlying traditional respiratory exercises in pulmonary rehabilitation and to provide evidence-based exercise protocols and appropriate dosing regimens for home-based rehabilitation in stable patients with COPD, thereby demonstrating clinical relevance and potential for broader application.

## Supplementary material

10.2196/91283 Checklist 1 SPIRIT checklist.

10.2196/91283 Checklist 2 TIDieR-checklist.

## Abbreviation

6MWT: 6-minute walk test
COPD: chronic obstructive pulmonary disease
CR-10: Category-Ratio 10
EMG: electromyography
FEV1: forced expiratory volume in one second
FVC: forced vital capacity
GOLD: Global Initiative for Chronic Obstructive Lung Disease
IKMT: isokinetic muscle strength test
MCID: minimal clinically important difference
PFT: pulmonary function test
sEMG: surface electromyography
SENIAM: Surface Electromyography for the Non-Invasive Assessment of Muscles
SGRQ: St. George’s Respiratory Questionnaire
